# A clinical retrospective study of implant as a risk factor for medication-related osteonecrosis of the jaw: surgery vs loading?

**DOI:** 10.1186/s40902-023-00398-2

**Published:** 2023-09-14

**Authors:** Yong-Dae Kwon, Hyunmi Jo, Jae-Eun Kim, Joo-Young Ohe

**Affiliations:** 1 Department of Oral and Maxillofacial Surgery, School of Dentistry, Kyung Hee University, Seoul, South Korea; 2 Department of Oral and Maxillofacial Surgery, Kyung Hee University Medical Center, Seoul, South Korea

**Keywords:** Medication-related osteonecrosis of the jaw, Implantation, Implant loading

## Abstract

**Background:**

Risk factors for developing medication-related osteonecrosis of the jaw (MRONJ) include the general condition of the patient, smoking habit, poor oral hygiene, and the type, duration, and administration route of the drug, dentoalveolar surgery, such as implant placement. This study aimed to discuss whether implants may induce osteonecrosis in older patients receiving long-term medication and to analyze the radiological pattern of the bone necrosis.

**Methods:**

This study included 33 patients diagnosed with dental implant-associated medication-related osteonecrosis of the jaw. Data regarding the medical history, type of medication used, durations of administration, laboratory test results, onset of bone necrosis since implant placement, type of opposing teeth, and radiological pattern of the bone necrosis on cone-beam computed tomography were recorded in patients with and without implants.

**Results:**

The most commonly used drug was bisphosphonate, with an average duration of use of 61.37 (± 53.72) months. The laboratory results showed average serum C-terminal cross-linking telopeptide (CTX) level of 0.23 ng/mL, vitamin D, 23.42 ng/mL, and osteocalcin, 4.92 ng/mL. Osteonecrosis occurred after an average of 51.03 (± 39.75) months following implant placement. Radiological evaluation revealed obvious sequestration in the implant-absent group, and the formation of a unit sequestration with an implant fixture (en bloc) was observed in the implant-present group. The patients underwent surgical treatment of sequestrectomy and explantation.

**Conclusion:**

Implant placement, especially loading, may be considered a potential risk factor for the development of osteonecrosis in patients undergoing antiresorptive treatment.

## Background

Antiresorptive agents, including bisphosphonates and denosumab, are commonly used to manage osteoporosis, osteopenia, and malignancy. This type of medication has been associated with medication-related osteonecrosis of the jaw (MRONJ), and patients with a history of using these medications experience osteonecrosis of the upper or lower jaw after dental surgical procedures [1]. The American Association of Oral and Maxillofacial Surgeons (AAOMS) changed the nomenclature for bisphosphonate-related osteonecrosis of the jaw to MRONJ in 2022 [2]. This emphasizes that osteonecrosis cases are not only associated with bisphosphonates but also with other types of antiresorptive agents. Although the disease pathogenesis has not been fully understood, it is strongly related to the drug function of inhibiting osteoclastic bone resorption and bone remodeling, and infection correlates with pre-existing dental disease. The risk factors for developing MRONJ include medication-related factors (type of medication and its duration and route of administration), systemic factors (sex, age, underlying diseases, smoking habit, and other comorbid conditions), and dentoalveolar surgeries such as tooth extraction or dental implant placement [3]. Several studies have shown that implants can be considered a local risk factor for MRONJ [3–5]. Particularly, bisphosphonates may contribute significantly to implant failure, and osteonecrosis can occur even around successfully osseointegrated implants [5]. Implant placement procedure is expected to be widely performed due to increasing life expectancy and higher efforts for oral rehabilitation [6], resulting in an increase in implant-associated MRONJ cases. This retrospective study aimed to assess whether implants are a risk factor for MRONJ and to evaluate the specific radiological patterns using cone-beam computed tomography (CBCT).

## Methods

### Patient selection

This study enrolled 33 patients diagnosed with implant-associated MRONJ according to their clinical and radiological evaluations between January 2012 and December 2019 at the Department of Oral and Maxillofacial Surgery at Kyung Hee University Dental Hospital. MRONJ was diagnosed according to the AAOMS guidelines [2]. The inclusion criteria were current or previous treatment with antiresorptive or antiangiogenic agents and exposed bone or bone that could be probed through an intraoral or extraoral fistula in the maxillofacial region, which was persistent for over 8 weeks. None of the patients had history of radiation therapy or obvious metastatic disease of the jaw. Data were collected from 33 patients who presented with MRONJ triggered by dental implants.

### Clinical evaluation

#### Patient evaluation

A comprehensive intraoral examination was performed for each patient to identify the necrotic sites. Medical records were analyzed to obtain their demographic data, prior medical history, and details of follow-up procedures. If the information from the patient statement was insufficient, a consultation paper could be issued to another hospital. The medication type (antiresorptives, steroids, and others) and the duration and route of administration were recorded. Regarding the causative implant, data regarding the location, interval between placement and initial symptom occurrence, and duration of functional loading were recorded. In these patients, the causative implants were exfoliated spontaneously owing to bone inflammation or had already been removed at other clinics before visiting our hospital. Blood tests were performed for bone turnover markers including C-terminal cross-linking telopeptide (CTx), osteocalcin, and vitamin D.

#### Radiologic evaluation

All patients underwent panoramic radiography and CBCT during their initial visit. Technetium-99 bone scintigraphy (bone scan) was performed if necessary. The radiographic parameters analyzed included the site, range of bone destruction, margins around the necrotic bone, and opposing teeth.

### Management of MRONJ

Patients who presented with a chief complaint of pain, swelling, or suppuration at the implant site were recommended to discontinue the medication or change to other types of drugs after consultation with the physician. Conservative treatment was preceded with wound dressing and administration of antibiotics, non-steroidal anti-inflammatory drugs, and 0.12% chlorhexidine gluconate oral rinse. The surgical procedure was performed when the symptoms did not improve or when obvious sequestration was observed. If the implant was adjacent to the necrotic bone or had lost stability, it was extracted in combination with the sequestrectomy procedure. Curettage or sequestrectomy of the necrotic bone was performed in cases where the implant was absent. These procedures were performed under general or local anesthesia.

### Statistical analyses

Mean values with standard deviations (SD) were calculated. *T*-test was performed using Statistical Product and Service Solutions, version 25 (IBM Corp., Armonk, NY, USA); *p*-values < 0.05 were considered statistically significant.

## Results

### Clinical features

#### Demographic data and medication information

**Fig. 1:**
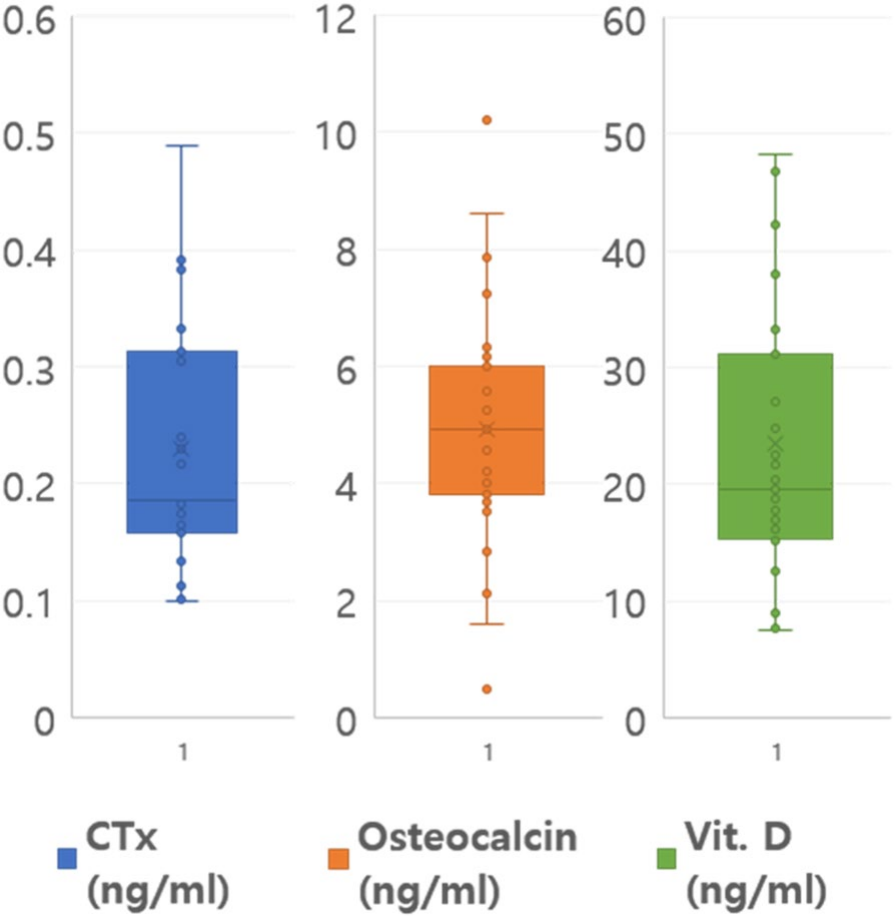
Laboratory test results

This study included 33 patients (2 males and 31 females; mean age, 74 years; SD, 6.92; range, 58–87 years) (Table 1). The indications for medication administration were osteoporosis in 32 patients and breast cancer in 1 patient. Three patients with osteoporosis also exhibited rheumatism. The number of oral bisphosphonate users was more than that of intravenous bisphosphonate users, and alendronate was the most commonly used drug. The mean duration of antiresorptive drug usage was 61.37 (± 53.72) months. The blood tests revealed the following results: CTx, 0.229 (± 0.103) ng/dL; osteocalcin, 4.917 (± 2.080) ng/dL; and vitamin D, 23.417 (± 11.521) ng/dL. Most patients were in the low-risk group based on their CTx values (Fig. 1).

**Table 1:** Demographic data of the participants

Variables	*N* (%), mean ± standard deviation (range)
Sex	
Male	2 (6.1)
Female	31 (93.9)
Age (years)	
58–69	7 (21.2)
70–79	18 (54.5)
80–89	8 (24.2)
Indication for medication use	
Osteoporosis	32 (96.9)
Rheumatism	3 (9.1)
Breast cancer	1 (3.0)
Medical history	
HTN	12 (36.4)
DM	2 (6.1)
Cardiovascular disease	3 (9.1)
Stroke	3 (9.1)
Number of residual teeth	15.88 ± 6.92 (2–28)
Number of implants	4.64 ± 3.55 (1–14)
Area of MRONJ	
Maxilla	2 (6.0)
Mandible	31 (94.0)

*HTN* Hypertension, *DM* Diabetes mellitus, *MRONJ* Medication-related osteonecrosis of the jaw

#### Associated factor and management of implant-associated MRONJ

In total, 47 implants were associated with MRONJ in this study. The MRONJ lesions were located in the mandible in 31/33 patients and in both the maxilla and mandible in 2/33 patients. Moreover, 28 implants of 22 patients had already been removed before visiting our hospital (Table 2).

**Table 2:** Details regarding medications and surgical procedures

	Duration of medication therapy		Total
	< 48 months	≥ 48 months	
Drugs			
Alendronate	8 (44.4)	6 (37.5)	14
Ibandronate	1 (5.6)	7 (43.7)	8
Zoledronate	2 (11.1)	-	2
Risedronate	3 (16.7)	1 (6.2)	4
Denosumab	2 (11.1)	-	2
Methotrexate	1 (5.6)	1 (6.2)	2
Steroids	1 (5.6)	1 (6.2)	2
Route			
Per oral	13 (72.2)	13 (86.7)	26
Intravenous	5 (27.8)	-	5
Per oral + intravenous	-	2 (13.3)	2
Surgical procedure			
Sequestrectomy	2 (11.1)	3 (20.0)	5
Explantation	4 (22.2)	4 (26.7)	8
Sequestrectomy + explantation	11 (61.1)	7 (46.7)	18
Debridement	1 (5.6)	1 (6.7)	2

*NSAIDs *Non-steroidal anti-inflammatory drugs

Sequestrectomy was performed under general anesthesia and local anesthesia in 8 and 22 patients, respectively. Some of the adjacent teeth were extracted when the necrosis spread. Eight patients (19 implants) underwent extraction at our hospital. The surgical procedure of explantation combined with sequestrectomy was performed under local or general anesthesia. Only two patients with recurrent MRONJ were treated surgically twice, while the others showed good outcomes with wounds covered by intact mucosa.

#### Implantation and development of implant-associated MRONJ

The associated factor of the MRONJ had been investigated (Table 3). Most of the patients were transferred from local dental clinics. Eleven patients developed osteonecrosis within 7 months after implantation, and 22 patients developed osteonecrosis after implant-loading. The dentists who referred the patients describe the time of initiation of osteonecrosis. In these 33 patients, the patients’ implants were functional at the beginning of loading, but symptoms gradually developed over time. The mean time interval between implant placement and initial symptom occurrence was 51.03 (± 39.75) months.

**Table 3:** Comparison of occurrence of MRONJ after implant placement and implant loading

		Osteonecrosis development	
		After implantation	After implant loading
Implant-associated MRONJ duration	< 36 months	4	11
	≥ 36 months	7	11

*MRONJ *Medication-related osteonecrosis of the jaw

### Radiographic features

Through CBCT evaluation, the tendency for bone necrosis was evaluated when the implant was present and absent (Table 4). The patients were divided into two groups according to the presence or absence of implants. During the first visit, 21 patients had already lost their implants (implant-absent cases), whereas 12 had retained their implants (implant-present cases).

**Table 4:** Radiographic features of implant-associated medication-related osteonecrosis of the jaw

	Implantation		Total
	Before drug therapy	After drug therapy	
Necrosis type			
Implant-present cases			
Osteolytic	2 (13.3)	3 (16.7)	5
En bloc	4 (26.7)	3 (16.7)	7
Implant-absent cases			
Diffuse margins	3 (20.0)	3 (16.7)	6
Sequestration	6 (40.0)	9 (50.0)	15
Opposing teeth			
Natural tooth	6 (40.0)	3 (16.7)	9
Prosthesis (Crown)	3 (20.0)	8 (44.4)	11
Implant	4 (26.7)	5 (27.8)	9
None	2 (13.3)	2 (11.1)	4

**Fig. 2:**
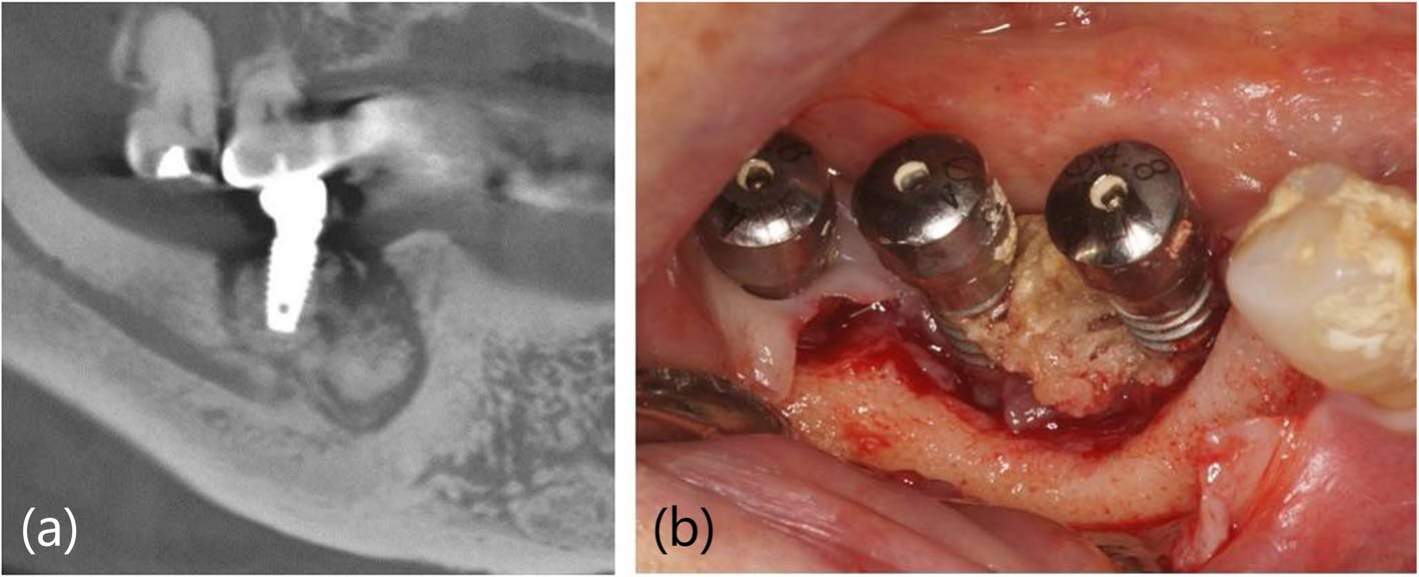
Necrotic bone sequestrated as a block including the implant in en bloc type. **a** Computed tomography image. **b** Clinical image

**Fig. 3:**
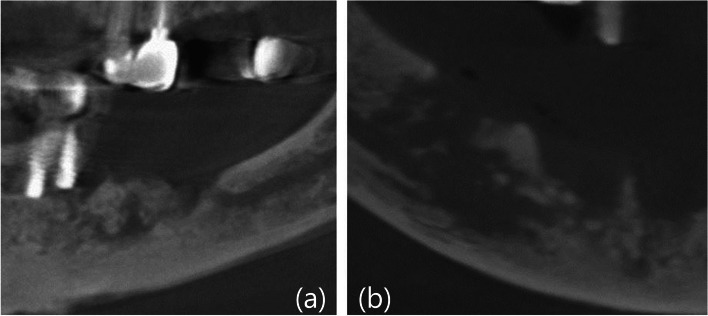
Obvious sequestration (**a**) and diffuse margins of necrotic bone (**b**) observed in implant-absent cases

In the implant-present cases, en bloc type of implant surrounded by sequestration was observed (*n* = 7/12), with the same incidence observed for diffuse margins of necrosis (Fig. 2). The characteristic bone necrosis pattern with implants was observed, wherein the necrotic bone surrounds the implant fixture. The most common opposing teeth were natural teeth, followed by implants. In the implant-absent cases, the incidence of evident sequestrae was higher than that of diffuse margins of bone necrosis (15 vs. 6) (Fig. 3).

### Statistical evaluation

We performed *t*-test to evaluate the comparison of implant-MRONJ period according to the timing of implant placement based on medication intake. The average duration of implant-associated MRONJ was significantly shorter in the patients undergoing implant placement after medication therapy than in those undergoing implantation before medication therapy (*p* < 0.05) (Table 5). However, this study has a limitation that the number of cases included is not sufficient for analysis.

**Table 5:** Comparing implant-associated medication-related osteonecrosis of the jaw duration by implantation time based on medication initiation

		Implant-associated MRONJ duration			
		*N*	Mean ± standard deviation	*t*	*p*
Implantation	Before medication initiation	15	80.87 ± 51.81	2.945	0.008
	After medication initiation	18	37.33 ± 26.67		

*MRONJ *Medication-related osteonecrosis of the jaw

## Discussion

Antiresorptives are the commonly prescribed medications to treat osteoporosis in older patients, a condition that weakens the bones and makes them prone to fractures. Antiresorptives work by reducing the rate at which the bone breaks down, which can help prevent further bone loss and reduce the risk of fracture [7].

In older patients with implants, such as dental implants or joint replacements, there is some concern that bisphosphonate use may increase the risk of implant failure [8]. Antiresorptives can affect the bone remodeling process, which is necessary for the implant to integrate properly with the surrounding bone [9, 10].

However, the evidence on this topic is controversial. Some studies found an increased risk of implant failure in patients taking bisphosphonates, whereas others did not. Overall, the risk appears to be relatively low, and many experts agree that the benefits of bisphosphonate treatment for osteoporosis outweigh its potential risks [11–13].

The multifactorial background of implant complications and failures has been extensively reviewed [14–17]. Recognizing jaw conditions that increase the risk of failure will allow the surgeon to make informed decisions and refine the treatment plan to optimize the outcomes. The success or failure of implants is closely related to the patient’s general condition, especially in cases of diabetes and antiresorptive therapy [18]. The effect of antiresorptive medicines on the osseointegration and survival of dental implants remains controversial. Previous studies have recommended that treatment with oral bisphosphonates is not a definite contraindication for dentoalveolar surgery. Recently, many cases of MRONJ associated with implant or invasive dental procedures have been reported [2]. This study aimed to investigate implants as the risk factor for MRONJ and to depict the clinical and radiological features of implant-associated MRONJ. We hypothesized that implant placement or loading is associated with MRONJ and analyzed various clinical factors. However, the results of these investigations were inconclusive.

The results of this study show that the time between functional loading and placement of implants and the onset of osteonecrosis can be long. In the literature review, the duration ranged from 44.4 to 89.6 months. Previous studies are of the view that treatment with oral bisphosphonates is not considered an absolute contraindication for dentoalveolar surgery, and implant placement does not need a “drug holiday” [19]. However, some studies have reported about patients with “delayed osteonecrosis” who had implants and were prescribed antiresorptive agents [4, 5]. A study analyzing 12 clinical cases reported that one patient developed osteonecrosis immediately after implant placement (2 months). For the remaining 11 patients, the specific time from implant placement to symptom onset was not analyzed [20]. In a similar study, Kwon et al. referred to the term “implant surgery triggered osteonecrosis” to define cases in which osteonecrosis occurred within 6 months after implant placement surgery. In their report, 58% of the cases with osteonecrosis were not related to implant insertion [5]. Most previous studies on peri-implant MRONJ showed no association with implant surgery; however, these cases were triggered by previously osseointegrated implants [21].

The results of our clinical study confirm that MRONJ with implants occurs more frequently after loading. Among the 33 patients, 11 patients had implant-surgery-triggered MRONJ, while 22 patients had implant loading-triggered MRONJ. Because MRONJ patients usually have low bone mineral density, loading of the occlusal force after osseointegration may lead to microfractures in the bone. With the onset of such microfractures, the sequestration grows in the form of a block in the presence of an implant (complete necrosis of the bone around the implant). In the other cases, extensive osteolysis around the implant with or without sequestration occurred similar to the pattern of implant failure. These types of MRONJ with implants can occur simultaneously, depending on the quality of the local bone.

It is important for patients taking antiresorptives or denosumab to inform their dentist or oral surgeon regarding their medication before undergoing any dental procedures, including implant placement. This allows the dentist or oral surgeon to take appropriate precautions and minimize the risk of complications, including MRONJ. In some cases, it may be advisable to delay or avoid implant placement altogether, especially in patients at a high risk of MRONJ.

## Conclusion

The average duration of implant-associated MRONJ was significantly shorter in the patients undergoing implant placement after medication therapy than in those undergoing implantation before medication therapy. Additionally, the incidence of MRONJ after implant loading was significantly higher than that after implant placement. Therefore, implant placement, especially loading, may be considered a potential risk factor for the development of osteonecrosis in patients undergoing antiresorptive treatment. The cause of this type of sequestration is not fully understood yet; therefore, the possible pathogenesis needs to be investigated in future studies.

## Data Availability

Data may be available on request due to privacy/ethical restrictions: The data that support the findings of this study may be available on request from the corresponding author. The data are not publicly available due to privacy or ethical restrictions.

## Abbreviations

MRONJ: Medication-related osteonecrosis of the jaw
CBCT: Cone-beam computed tomography
AAOMS: American Association of Oral and Maxillofacial Surgeons
CTx: C-terminal cross-linking telopeptide
